# Limited role of DWI with apparent diffusion coefficient mapping in breast lesions presenting as non-mass enhancement on dynamic contrast-enhanced MRI

**DOI:** 10.1186/s13058-019-1208-y

**Published:** 2019-12-04

**Authors:** Daly Avendano, Maria Adele Marino, Doris Leithner, Sunitha Thakur, Blanca Bernard-Davila, Danny F. Martinez, Thomas H. Helbich, Elizabeth A. Morris, Maxine S. Jochelson, Pascal A. T. Baltzer, Paola Clauser, Panagiotis Kapetas, Katja Pinker

**Affiliations:** 1 Department of Radiology, Breast Imaging Service, Memorial Sloan Kettering Cancer Center, Suite 705, 300 E 66th Street, New York, NY 10065 USA; 2 Department of Breast Imaging, Breast Cancer Center TecSalud, ITESM Monterrey, Monterrey, Nuevo Leon Mexico; 3 Department of Biomedical Sciences and Morphologic and Functional Imaging, University of Messina, Messina, Italy; 4 Department of Diagnostic and Interventional Radiology, University Hospital Frankfurt, Frankfurt, Germany; 5 Department of Medical Physics, Memorial Sloan Kettering Cancer Center, 1275 York Ave, New York, NY 10065 USA; 6 Division of Molecular and Gender Imaging, Department of Biomedical Imaging and Image-Guided Therapy, Medical University of Vienna, Vienna, Austria

**Keywords:** Breast cancer, Magnetic resonance imaging, Non-mass enhancement, Diffusion-weighted imaging

## Abstract

**Background:**

Available data proving the value of DWI for breast cancer diagnosis is mainly for enhancing masses; DWI may be less sensitive and specific in non-mass enhancement (NME) lesions. The objective of this study was to assess the diagnostic accuracy of DWI using different ROI measurement approaches and ADC metrics in breast lesions presenting as NME lesions on dynamic contrast-enhanced (DCE) MRI.

**Methods:**

In this retrospective study, 95 patients who underwent multiparametric MRI with DCE and DWI from September 2007 to July 2013 and who were diagnosed with a suspicious NME (BI-RADS 4/5) were included. Twenty-nine patients were excluded for lesion non-visibility on DWI (*n* = 24: 12 benign and 12 malignant) and poor DWI quality (*n* = 5: 1 benign and 4 malignant). Two readers independently assessed DWI and DCE-MRI findings in two separate randomized readings using different ADC metrics and ROI approaches. NME lesions were classified as either benign (> 1.3 × 10^−3^ mm^2^/s) or malignant (≤ 1.3 × 10^−3^ mm^2^/s). Histopathology was the standard of reference. ROC curves were plotted, and AUCs were determined. Concordance correlation coefficient (CCC) was measured.

**Results:**

There were 39 malignant (59%) and 27 benign (41%) lesions in 66 (65 women, 1 man) patients (mean age, 51.8 years). The mean ADC value of the darkest part of the tumor (Dptu) achieved the highest diagnostic accuracy, with AUCs of up to 0.71. Inter-reader agreement was highest with Dptu ADC max (CCC 0.42) and lowest with the point tumor (Ptu) ADC min (CCC = − 0.01). Intra-reader agreement was highest with Wtu ADC mean (CCC = 0.44 for reader 1, 0.41 for reader 2), but this was not associated with the highest diagnostic accuracy.

**Conclusions:**

Diagnostic accuracy of DWI with ADC mapping is limited in NME lesions. Thirty-one percent of lesions presenting as NME on DCE-MRI could not be evaluated with DWI, and therefore, DCE-MRI remains indispensable. Best results were achieved using Dptu 2D ROI measurement and ADC mean.

## Background

Although dynamic contrast-enhanced magnetic resonance imaging (DCE-MRI) of the breast is the most sensitive method for the detection of breast cancer, it is limited in assessing the likelihood of malignancy for non-mass enhancement (NME) breast lesions [1–5], resulting in unnecessary breast biopsies [6–9]. With advances in imaging techniques and hardware, such as better gradient systems and multichannel coils, DWI with ADC mapping has emerged as the most robust and reliable adjunct to DCE-MR with reported sensitivities of up to 96% and specificities of up to 100% for their combination [4, 10–13]. In addition, with recent concerns about the safety of gadolinium-containing contrast agents [14], DWI with ADC mapping has been suggested as an alternative unenhanced technique for breast cancer screening and diagnosis [15–19]. However, the majority of the available data for DWI is for enhancing masses, and concerns remain that DWI may be less sensitive and specific in the assessment of NME lesions [20]. Additionally, while it has been shown that a 2D region of interest (ROI) ADC measurement approach in the enhancing tumor with the visually assessed lowest ADC is the most practical and diagnostically accurate measurement in mass lesions [21–23], the best and most reliable measurement in NME lesions remains unclear. To close these gaps in knowledge, the aim of this study was to assess the diagnostic accuracy of DWI using different ROI measurement approaches and ADC metrics in breast lesions presenting as NME lesions on DCE- MRI and to assess inter-reader agreement and repeatability of ADC measurements.

## Methods

The local institutional review board approved this prospective single-institution study (EK 510/2009) and retrospective data analysis. The research was performed in accordance with relevant guidelines/regulations, and informed consent was obtained from all patients prior to multiparametric MRI of the breast.

### Patients

A prospectively and consecutively populated research database was searched for patients who underwent multiparametric MRI of the breast with DCE and DWI between September 2007 and July 2013 and who fulfilled the following inclusion criteria: 18 years or older; not pregnant; not breastfeeding; no previous breast cancer treatment; presence of NME (BI-RADS 4–5) on DCE-MRI suspicious according to BI-RADS lexicon, i.e., unilateral with segmental, focal, or linear distribution; and no contraindications for MRI or MRI contrast agents. Patients underwent breast MRI to evaluate the following conditions: (a) equivocal findings on conventional imaging (BIRADS 0), (b) suspicious lesions or lesions highly suggestive of malignancy on conventional imaging (BIRADS 4 and 5), and (c) preoperative staging of biopsy-proven breast cancer (BI-RADS 6).

We identified 95 patients who fulfilled these criteria. Of these, 29 patients were excluded for the following reasons: (a) lesion not visible on DWI and ADC map (*n* = 24: 12 benign and 12 malignant) and (b) poor DWI quality (*n* = 5: 1 benign and 4 malignant). Among the lesions that were not visible on DWI, there were 8/12 ductal carcinomas in situ (DCIS), 3/12 invasive lobular carcinomas (ILC), and 1/12 invasive ductal carcinomas (IDC). Among the lesions with poor DWI quality, there were 2/4 DCIS and 2/4 IDC.

**Fig. 1:**
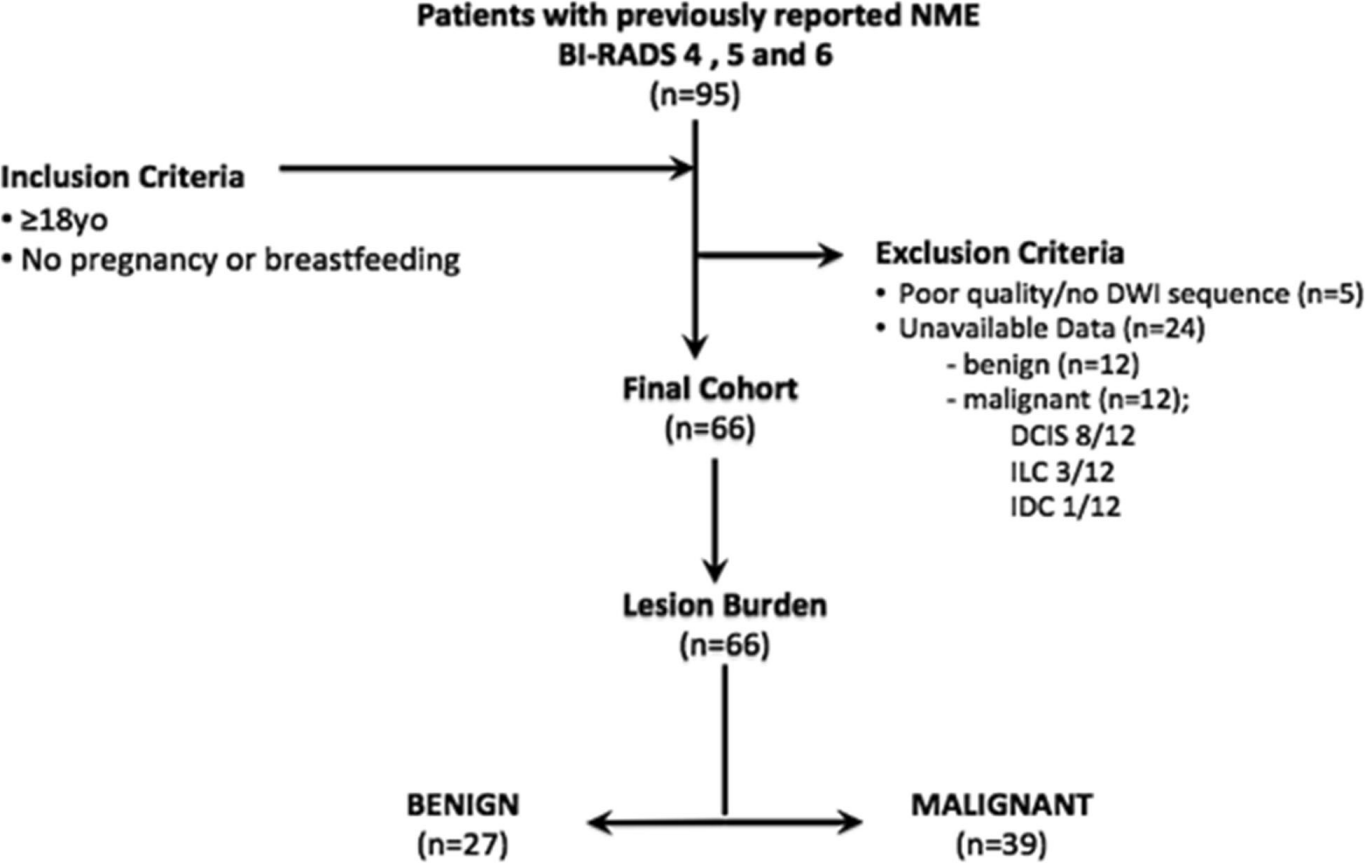
The scheme summarizes the data selection criteria of our study inclusion and exclusion criteria. BI-RADS, Breast Imaging Reporting and Data System; DCIS, ductal carcinoma in situ; DWI, diffusion-weighted imaging; IDC, invasive ductal carcinoma; ILC, invasive lobular carcinoma; NME: non-mass enhancement

Therefore, 66 patients were included for analysis. Patient selection is detailed in Fig. 1. Electronic medical records were reviewed to record patient age as well as histopathology results which included tumor grade, subtype, and receptor status for malignant lesions.

A number of patients included in this study have been analyzed and reported before in a different context [6]. In the prior study, the authors developed a BI-RADS®-adapted reading for multiparametric MRI of the breast using DCE-MRI and DWI that adapted ADC thresholds to the assigned BI-RADS® classification and assessed the diagnostic value of this BI-RADS®-adapted reading by an objective comparison with previously published assessment methods in patients with both mass (*n* = 255) and NME (*n* = 36) breast lesions.

The current study includes a larger patient number and focuses solely on the diagnostic value of DWI in NME lesions, including the 36 NME breast lesions that have been reported before.

### Magnetic resonance imaging technique

All patients underwent 3 T MRI (Tim Trio, Siemens, Erlangen, Germany) in the prone position using a 4-channel breast coil (In Vivo, Orlando, FL, USA). In premenopausal women, MRI was performed in the second week of the menstrual cycle. Details on the MRI protocol have been previously published [24]. The DWI protocol included axial three-acquisition trace diffusion-weighted, double-refocused, single-shot echo-planar imaging with inversion recovery fat suppression (TR/TE/TI 13,700/83/220 ms; FOV 340 × 117 mm; 40 slices at 3.5 mm; matrix 192 × 64 (50% oversampling); two *b* values of 50 and 850 s/mm^2^; bandwidth 1446 Hz/pixel; 3:19 min).

### Data analysis

Two breast radiologists (DA, MAM), each with more than 3 years of experience in interpretation of breast MRI, independently evaluated the DW images and corresponding ADC maps. Both readers were aware that patients had a breast lesion, but they were not provided with previous imaging or histopathological results. Each reader performed all the readings twice, with a washout period of at least 3 weeks.

**Fig. 2:**
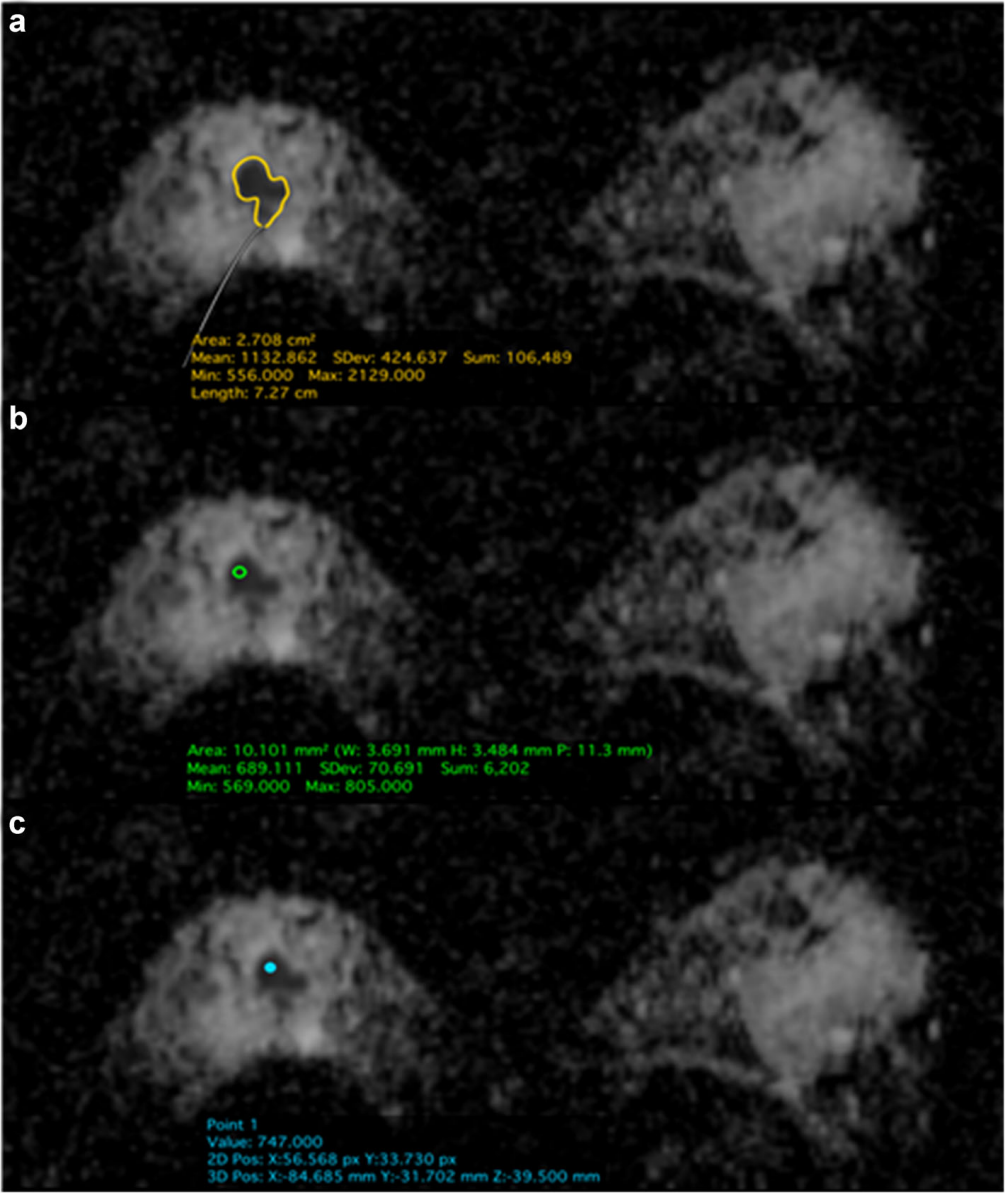
Invasive ductal carcinoma, grade 2, in a 47-year-old patient undergoing preoperative MRI. Apparent diffusion coefficient (ADC), axial views (**a**–**c**). The images show a hypointense area of restricted diffusion in the central part of the right breast. Examples of the three methods used to measure the ADC values: **a** whole tumor delineation, **b** darkest part of the tumor delineation, and **c** point tumor delineation. The three regions of interest show low ADC values < 1.3 × 10^−3^ mm^2^/s indicating that the enhancement is highly suspicious of malignancy (Breast Imaging Reporting and Data System 5)

All Digital Imaging and Communications in Medicine (DICOM) images were loaded onto the open-source image processing tool OsiriX (OsiriX Foundation, Geneva, Switzerland). The readers evaluated the lesions on both DW images and ADC maps and then recorded mean ADC values on ADC maps using three measurement approaches: (a) whole tumor (Wtu) ROI, (b) darkest part (Dptu) tumor 10 mm ROI, and (c) point tumor (Ptu) 3 mm ROI. For Wtu delineation, the readers segmented the entire 3D volume of the lesion by contouring the borders for each slice. For Dptu delineation, the readers used a *10-mm 2D ROI* in the visually darkest (i.e., most suspicious) region of the enhancing tumor [9]. A similar approach was used for Ptu delineation by placing a 2D ROI *point tool* on the darkest part of the lesion (Fig. 2).

The mean, minimum, and maximum ADC values were recorded for Wtu and Dptu while only one ADC value was obtained for the Ptu.

A threshold of 1.3 × 10^−3^ mm^2^/s for the ADC value was used as the cutoff for the differentiation between benign (> 1.3 × 10^−3^ mm^2^/s) and malignant (≤ 1.3 × 10^−3^ mm^2^/s) lesions [11, 25, 26].

### Histopathology

Histopathological diagnosis was established using image-guided needle biopsy or surgery no later than 1 week after MRI. In the case of a benign diagnosis at image-guided needle biopsy, the final diagnosis was benign. In the case of a high-risk lesion with uncertain potential for malignancy, the final diagnosis was established with surgery.

### Statistical analysis

All calculations were performed on a per-lesion basis. Univariate analysis was performed. Differences in imaging features between malignant and benign lesions were assessed using the Wilcoxon signed-rank test. ROC curves were plotted, and the area under the curve (AUC) was determined. *p* values of ≤ 0.5 were considered significant.

To assess intra- and inter-reader agreement, concordance correlation coefficient (CCC) was used on the continuous measures (ADC values) obtained by the two independent readers. The CCC provides a measure of both precision and accuracy in relation to the line of perfect concordance (45° line on a scatterplot). The better the agreement between the two readers for the parameter, the closer the coefficient will be to 1. The Bland–Altman assessment was used to compare the absolute difference as a percentage of the average of reads, including 95% limits of agreement.

## Results

### Lesions characteristics

There were 66 lesions in 66 patients (65 women, 1 man; mean age 51.8 ± 10.8 years (range 26–76 years), Table 1). Histopathology revealed 39 malignant (59%) and 27 benign (41%) lesions. The mean size of all lesions, as measured on DCE-MRI, was 27.8 ± 18.3 mm (range 5–80 mm).

**Table 1:** Baseline characteristics of study population

	*N* (%)	Mean age (years)	Standard deviation (SD)	Range (years)
Total of the study population	66 (100)	51.8	± 10.8 years	26–76
Women	65 (98.5)	51	± 11.1 years	26–76
Men	1 (1.5)	67	–	–
Premenopausal women	32 (49)	42	± 6 years	26–49
Postmenopausal women	33 (51)	60	± 7.5 years	50–76

All lesions were seen on both sequences DW images and ADC map.

For a detailed description of the histopathological diagnosis and mean lesion size, see Table 2.

**Table 2:** Detailed histopathological diagnosis of all malignant and benign non-mass enhancement lesions

Histopathology	*N* (%)	Mean size (mm)	Standard deviation (mm)	Range (mm)
Non-mass enhancement lesions	66 (100)	40	25	5–98
Malignant	39/66 (59)	48.4	26	5–98
Ductal carcinoma in situ	4 (10)	33.5	33	7–89
Invasive ductal carcinoma	24 (62)	48	27	5–98
Invasive lobular carcinoma	9 (23)	56.5	20	25–85
IDC + DCIS	1 (2.5)	30	–	–
IDC + LCIS	1 (2.5)	60	–	–
Benign	27/66 (41)	28	18	5–80
FA/FAH	5 (19)	21.5	11	10–38
Adenosis, sclerosing adenosis, focal fibrosis, apocrine metaplasia, breast parenchyma, fibrocystic changes	12 (44)	23	16	5–50
Papilloma	1 (4)	45	–	–
High-risk (CCC with atypia, papilloma with atypia)	2 (7)	42	3	40–44
Other (chronic abscess, gynecomastia, fat necrosis, scar tissue)	7 (26)	33.5	24	6–80

*IDC* invasive ductal carcinoma, *ILC* invasive lobular carcinoma, *DCIS* ductal carcinoma in situ, *LCIS* lobular carcinoma in situ, *FA* fibroadenoma, *FAH* fibro-adenomatoid hyperplasia, *CCC* columnar cell changes

### Differentiation of benign and malignant breast tumors

**Fig. 3:**
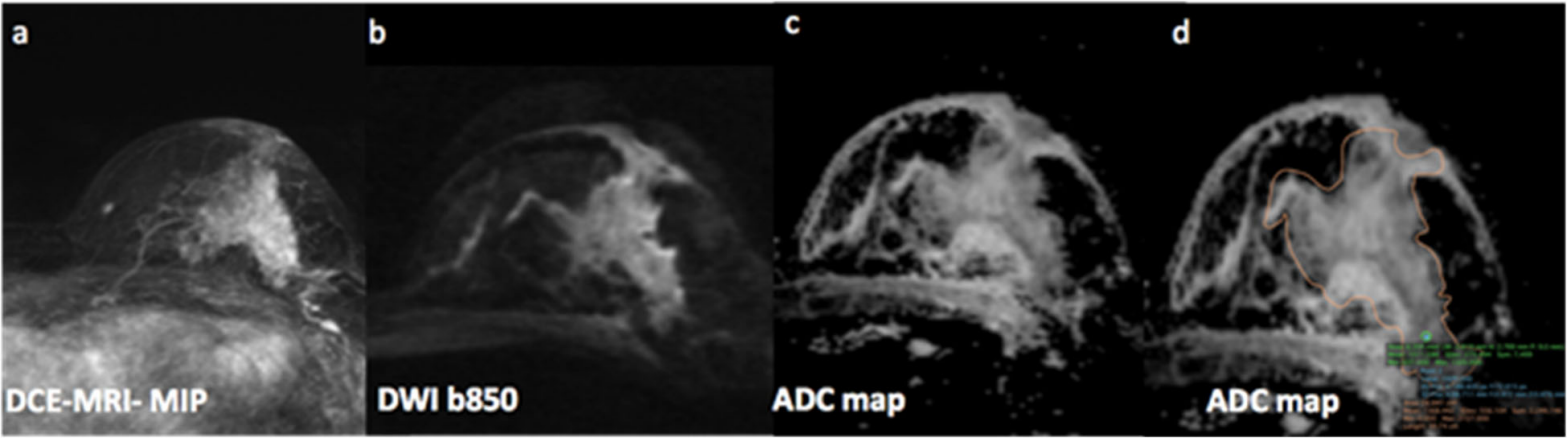
Invasive lobular cancer in a 49-year-old, with biopsy-proven invasive lobular cancer, grade 2. **a** Dynamic contrast-enhancement MRI maximum intensity projection of the left breast shows in the upper-outer quadrant, a 63-mm segmental area of non-mass enhancement, with heterogeneous internal enhancement pattern. **b** Diffusion-weighted sequence at b 850 and **c** apparent diffusion coefficient (ADC) map. **d** Among the three segmentation tools, the darkest part of the tumor (Dptu) 2D region of interest (ROI) proved to yield the highest diagnostic accuracy, showing the lowest ADC values (Dptu ADC mean, 1.021 × 10^−3^ mm^2^/s; whole tumor ROI, 1.568 × 10^−3^ mm^2^/s; point tumor delineation ROI, 1.029 × 10^−3^ mm^2^/s)

Results show that the diagnostic accuracy of DWI with ADC mapping is limited in lesions presenting as NME lesions on DCE-MRI regardless of the ROI measurement approach and different ADC metrics used. The Dptu ADC mean measurement approach most consistently showed differences in the ADC values of benign and malignant with the best AUC of 0.71 (Fig. 3).

Several ROI placement approaches were found to yield lower ADC values for malignant NME breast lesions than for benign NME breast lesions for at least one reader in one reading: Wtu ADC max (reading 1, reader 1, *p* = 0.01), Dptu ADC max (reading 2, reader 1, *p* = 0.08), Wtu ADC mean (reading 2, reader 2, *p* = 0.02), Wtu ADC min (reading 2, reader 2, *p* = 0.03), Dptu ADC min (reading 2, reader 2, *p* = 0.02), and Ptu ADC mean (reading 2, reader 2, *p* = 0.001).

ADC values for all lesions and *p* values for the three different ROI placement approaches, stratified by benign and malignant histopathological diagnosis, are shown in Table 3. The AUC derived from both readers for the differentiation between benign and malignant NME breast lesions and readings for all measurement approaches are summarized in Table 4. ROC curves are provided in Additional file 1: Figure S1, Additional file 2: Figure S2, and Additional file 3: Figure S3).

**Table 3:** ADC values for all lesions and *p* values for the three different ROI placement approaches

ADC metric (× 10^−3^ mm^2^/s)	Benign (*n* = 27)	Malignant (*n* = 39)	Reading 1 (*p* values)	Benign (*n* = 27)	Malignant (*n* = 39)	Reading 2 (*p* values)
Wtu ADC max (reader 1)	2.126 (0.254–2.988)	2.523 (198–4095)	0.01*	2.318 (1.930–3085)	2.311 (1.466–2.968)	0.66
Wtu ADC max (reader 2)	2.395 (1.881–3.568)	2.563 (1.620–4.095)	0.12	2.302 (1.552–3.069)	2.323 (1.649–2.974)	0.86
Wtu ADC mean (reader 1)	1.273 (0.118–1.898)	1.372 (0.129–2.668)	0.50	1.392 (1.004–1980)	1.281 (0.726–1.794)	0.19
Wtu ADC mean (reader 2)	1.377 (0.935–2028)	1.356 (0.846–2020)	0.68	1.462 (1.005–2.141)	1.273 (0.870–1.630)	0.02
Wtu ADC min (reader 1)	0.225 (0–1.671)	0.195 (0–1.071)	1	0.180 (0–0.731)	0.187 (0–1.188)	0.90
Wtu ADC min (reader 2)	0.137 (0–1.194)	0.099 (0–0.970)	0.11	0.356 (0–1.353)	0.140 (0–1.146)	0.03*
Dptu ADC max (reader 1)	1.654 (0.254–2.438)	1.607 (0.139–2.814)	0.60	1.711 (1.143–2.617)	1.469 (0–2.763)	0.08*
Dptu ADC max (reader 2)	1.702 (0.438–2.379)	1.494 (0.053–2.825)	0.02	1.721 (1.103–2.686)	1.501 (0.687–2.370)	0.03
Dptu ADC mean (reader 1)	1.249 (0.252–2.176)	1.201 (0.012–1.942)	0.85	1.306 (0.970–2.463)	1.122 (0.619–1.761)	0.048*
Dptu ADC mean (reader 2)	1.996 (0.303–2.009)	1.154 (0.044–2.363)	0.06*	1.442 (0.780–2.280)	1.183 (0.601–1.744)	0.004*
Dptu ADC min (reader 1)	0.824 (0–1.904)	0.913 (0–2.048)	0.17	0.916 (0–2.249)	0.818 (0–1.670)	0.35
Dptu ADC min (reader 2)	0.871 (0–2.059)	0.839 (0–1.994)	0.75	1.402 (0.120–9.191)	0.848 (0–1.476)	0.02
Ptu ADC mean (reader 1)	1.127 (0–1.946)	1.324 (0.200–6.876)	0.56	1.238 (0.470–1.963)	1.144 (0.540–2.174)	0.19
Ptu ADC mean (reader 2)	1.512 (0.281–10.005)	1.018 (0.284–1.942)	0.206	1.661 (0.808 10.003)	1.057 (0.496–1.571)	0.001*

*ADC* apparent diffusion coefficient, *Wtu*, whole tumor, *Dptu* darkest part of the tumor, *Ptu* point tumor

*Denotes a statistical significance

**Table 4:** Area under the curve for both readers, both readings, all measurement approaches, and ADC metrics

Parameters	Reading 1		Reading 2	
	R1	R2	R1	R2
Wtu ADC max	0.699 (*p* = 0.002)*	0.613 (*p* = 1.00)	0.467 (*p* = 0.65)	0.486 (*p* = 0.85)
Wtu ADC mean	0.549 (*p* = 0.51)	0.530 (*p* = 0.67)	0.596 (*p* = 0.17)	0.669 (*p* = 0.01)*
Wtu ADCmin	0.500 (*p* = 0.99)	0.599 (*p* = 0.67)	0.508 (*p* = 0.90)	0.646 (*p* = 0.02)*
Dptu ADC max	0.538 (*p* = 0.60)	0.667 (*p* = 0.02)*	0.628 (*p* = 0.07)	0.657 (*p* = 0.02)*
Dptu ADC mean	0.512 (*p* = 0.84)	0.638 (*p* = 0.05)*	0.644 (*p* = 0.03)*	0.709 (*p* = 0.001)*
Dptu ADC min	0.599 (*p* = 0.17)	0.523 (*p* = 0.75)	0.567 (*p* = 0.36)	0.669 (*p* = 0.01)
Ptu ADC mean	0.542 (*p* = 0.56)	0.592 (0.21)	0.595 (*p* = 0.19)	0.736 (*p* = 0.0002)*

*ADC* apparent diffusion coefficient, *Wtu* whole tumor, *Dptu* darkest part of the tumor, *Ptu* point tumor

### Inter- and intra-reader agreement

Inter- and intra-reader agreement in assessing ADC values was generally fair to moderate (Table 5 and Table 6). Inter-reader agreement was highest for Dptu ADC max (CCC = 0.420) and lowest for Ptu ADC min (CCC = − 0.014). Although both readers achieved the best intra-reader agreement with the Wtu measurement approach (CCC = 0.435 for reader 1, 0.412 for reader 2), this was not the most diagnostic accurate ADC measurement approach.

**Table 5:** Inter-reader agreement and concordance correlation coefficient for ADC measurements in reading 1 and reading 2

ADC metric	Reading 1		Reading 2	
	CCC (95% confidence interval)	Strength of agreement	CCC (95% confidence interval)	Strength of agreement
Wtu ADC max	0.35 (0.15, 0.55)	Fair	0.04 (− 0.01, 0.09)	Slight
Wtu ADC mean	0.34 (0.15, 0.54)	Fair	0.04 (0.32, 0.69)	Slight
Wtu ADC min	0.30 (0.10, 0.49)	Slight	0.09 (0.15, 0.32)	Slight
Dptu ADC max	0.141 (0.09, 0.38)	Slight	0.42 (0.23, 0.62)	Moderate
Dptu ADC mean	0.11 (− 0.11, 0.37)	Slight	0.32 (0.11, 0.53)	Fair
Dptu ADC min	0.13 (0.03, 0.19)	Slight	0.03 (− 0.13, 0.19)	Slight
Ptu ADC mean	− 0.01 (− 0.24, 0.22)	Slight	0.04 (− 0.09, 0.18)	Slight

*Wtu* whole tumor, *Dptu* darkest part of the tumor, *Ptu* point tumor, *max* maximum, *min* minimum

**Table 6:** Intra-reader agreement and 95% confidence intervals for reader 1 and reader 2 for all measured MRI parameters

ADC metric	Reader 1		Reader 2	
	Rho-c (95% confidence Interval)	Strength of agreement	Rho-c (95% confidence interval)	Strength of agreement
Wtu max ADC	0.37 (0.41, 0.74)	Fair	0.24 (0.07, 0.41)	Slight
Wtu mean ADC	0.44 (0.26, 0.61)	Moderate	0.41 (0.21, 0.62)	Moderate
Wtu min ADC	0.21 (− 0.02, 0.44)	Slight	0.29 (0.09, 0.48)	Slight
Dptu max ADC	0.18 (− 0.06, 0.41)	Slight	0.33 (0.11, 0.54)	Fair
Dptu mean ADC	0.39 (0.20, 0.59)	Fair	0.05 (− 0.01, 0.12)	Slight
Dptu min ADC	0.37 (0.41, 0.74)	Fair	0.24 (0.07, 0.41)	Slight
Ptu mean ADC	0.44 (0.26, 0.61)	Moderate	0.41 (0.21, 0.62)	Moderate

*ADC* apparent diffusion coefficient, *Wtu*, whole tumor, *Dptu* darkest part of the tumor, *Ptu* point tumor, *max* maximum, *min* minimum

The Bland–Atman plots for measured parameters are provided in Additional file 4: Figure S4, Additional file 5: Figure S5, and Additional file 6: Figure S6, showing the percent differences of the measurements between the two readers versus the average of the two readers’ measurements.

## Discussion

The results of the current study show that the diagnostic accuracy of DWI with ADC mapping is limited in lesions presenting as NME lesions on DCE-MRI regardless of the ROI measurement approach and different ADC metrics used. Up to a third of NMEs cannot be evaluated with DWI, and therefore, DCE-MRI is still indispensable for detection and characterization of NME lesions.

Previous studies investigated DWI with ADC mapping for the assessment of breast lesions, mainly masses, and only few NME with reported sensitivities of up to 96% and specificities of up to 100% [10–13, 22, 27, 28]. To the best of our knowledge, this is the largest cohort of NME lesions with DCE and DWI reported so far and our results confirm the suspicion that DWI with ADC mapping performs less well for the differentiation of benign and malignant breast lesions in NME lesions than in mass lesions. Across ROI measurement approaches and different ADC metrics used in this study, the diagnostic accuracy of DWI in NME lesion is moderate at best with AUCs ranging between 0.467 and 0.736. The Dptu ADC mean measurement approach seems to be the diagnostically yielding consistently AUCs of 0.71. In addition, it has to be noted that in our study, 31% (29/95) of lesions presenting as NME lesions on DCE-MRI could not be evaluated on DWI due to either non-visibility (*n* = 24: 12 benign and 12 malignant) or poor DWI quality (*n* = 5: 1 benign and 4 malignant). These non-visible lesions and lesions with poor DWI quality comprised not only benign entities but also 6 invasive breast cancers and 10 DCIS, highlighting that DWI alone misses breast cancer and that DCE-MRI is still indispensable for detection and characterization of NME lesions. Our findings expand on prior data in smaller NME series. Kul et al. [29] found that the effectiveness of DWI using ADC mean was lower for NME lesions as compared with masses and that the difference between the ADCs of benign and malignant lesions was smaller for NME lesions. Partridge et al. [30] reported that although DWI using a Wtu measurement approach and ADC mean is a promising tool for differential diagnosis for both masses and NME lesions, ADC measurements may be more useful for discriminating benign from malignant in masses than for discriminating lesions with NME enhancement. Recently, studies have investigated intravoxel incoherent motion and quantitative non-gaussian diffusion MRI [31–33]. It was demonstrated that higher *b* values may be a way to improve tumor-to-tissue contrast, lesion visibility, and image quality of DWI for the detection and characterization of breast tumors. However, to date, none of these studies have specifically focused on the diagnostically challenging NME lesions and the full potential of use of higher *b* values for improving diagnostic accuracy in NME lesions needs to be investigated in future studies.

We also aimed to answer the question of whether ADC measurements and ROI approaches other than those employed in prior studies may improve diagnostic accuracy of DWI in NME lesions. We found that the diagnostic accuracy of ADC measurements in NME lesions was significantly impacted by ROI choice and placement. Dptu ADC mean most consistently showed significant differences in ADC values of benign and malignant lesions, yielding the highest diagnostic accuracy. However, other ROI measurement approaches as well as different ADC metrics showed either less consistency and/or diagnostic accuracy, indicating less practicality for their clinical use. Our results are in agreement with Bickel et al. [34], who studied the influence of ROI placement and different ADC parameters on ADC values, diagnostic performance, inter-reader agreement, and measurement time in breast tumors, and who also found that ADC in NME had a lower accuracy compared with that achieved in mass lesions (AUC = 0.64–0.73 vs. 0.96–0.97).

To the best of our knowledge, the inter- and intra-reader agreement of different ADC measurement approaches and metrics for NME lesions have not been reported. Our study shows that ADC measurements between and within readers were only slight to moderate in agreement, which is not entirely unexpected in NME lesions. The measurement approach that yielded the highest diagnostic accuracy, i.e., Dptu, achieved only slight to fair inter- and intra-reader agreement, which is likely due to the placement of the ROIs after subjective radiologist’s review; therefore, not necessarily the same ROI location is chosen by each reader and for each reading. Moderate intra-reader agreement was achieved with both Wtu ADC mean measurement and Ptu ADC mean, but these were not the approaches with the highest diagnostic accuracy. The moderate agreement for Wtu ADC mean was most likely because NME lesions are often difficult to delineate, and therefore, the size and shape of ROI is more prone to variation even within readers. For Ptu, not necessarily the same point is chosen in each reading, leading to different ADC values. As for the intra-reader agreement, Dptu ADC max reached moderate intra-reader agreement. While the inter-reader agreement for Wtu ADC mean was more consistent between readers, it was not associated with the best diagnostic accuracy. Additionally, for the purpose of breast cancer diagnosis, a 2D ROI ADC mean measurement approach seems most practical.

Whereas this study focused solely on NME lesions, other studies have reported inter- and intra-reader agreement for DWI with ADC in lesions that included mostly masses. Bickel et al. [34] found that minimum ADC showed the best diagnostic performance (AUC 0.93–0.96), followed by mean ADC obtained from 2D ROIs (0.93–0.94), and both achieved high intra- (ICC 0.85–0.94) and inter-reader reproducibility (ICC 0.74–0.94). Median measurement time was significantly shorter for the 2D ROIs (*p* < 0.001). It should be noted that there were only 29 NME lesions in this patient population. Furthermore, when considering only NME lesions, the ADC achieved in their cohort was less accurate than in ours (AUC = 0.64–0.73). Spick et al. [27] led an intra-individual prospective clinical study of 40 consecutive patients with suspicious findings, including only 8 NME lesions. Reproducibility and repeatability showed high agreement for repeated examinations, readers, and measurements (all ICCs > 0.9, coefficient of variations 3.2–8%), indicating little variation. The Bland–Altman plots demonstrated no systematic differences, and diagnostic accuracy was not significantly different in the two repeated examinations (all ROC curves > 0.91, *p* > 0.05). There is consensus that reproducibility, repeatability, and diagnostic accuracy of DWI is necessary for its use as a potential quantitative imaging characteristic to enable improved breast lesion detection, characterization, and assessment of treatment response. Newitt et al. [35] evaluated the repeatability and reproducibility of breast tumor ADC in a multi-institution clinical trial setting, using standardized DWI protocols and quality assurance procedures. ADC repeatability was excellent in 80% (71/89) of cases. However, the authors did not report the number of NME lesions in their cohort. In contrast to other studies that reported inter- and intra-agreement for DWI with ADC mapping results for masses, our study demonstrated poorer reproducibility (fair to moderate). However, considering that our cohort consisted exclusively on NME lesions, this was expected.

Our study has some limitations. The sample size of our cohort is relatively small, but to the best of our knowledge, this is the largest cohort of NME lesions with DCE (*n* = 95) and DWI (*n* = 66, 24 not visible in DWI and 5 DWI quality insufficient) reported so far. This study was also conducted at a single tertiary center institution, and the interpretations were performed by experienced breast fellowship-trained radiologists, potentially making it difficult to extrapolate to community practice. Therefore, the overall rate of malignancy is high and the results might not be applicable to every radiologist, but it has to be noted that international guidelines [26] recommend that clinical breast MRI is reported by breast specialists. Another limitation is the retrospective nature of this study; therefore, the acquired different ADC measurements were not used in clinical decision-making. However, such retrospective studies are necessary to gather relevant information to allow future standardization and facilitate optimal clinical application implementation. Further, only BI-RADS 4 and 5 NMEs were included in the study and this could have caused a selection bias, leading to potential issues with statistical power, precision, and validity. Additional studies with larger cohorts are required to confirm our findings.

## Conclusions

In conclusion, the accuracy of DWI with ADC mapping is limited in breast tumors presenting as NME lesions, with best results being achieved using ADC mean and a 2D ROI measurement approach. Up to a third of NMEs cannot be evaluated with DWI, and therefore, DCE-MRI remains indispensable.

## Supplementary information

**Additional file 1: Figure S1.** ROC curves and AUC (in brackets) for the minimum, mean, and maximum ADC of the Whole Tumor 3D ROI segmentation approach.

**Additional file 2: Figure S2.** ROC curves and AUC (in brackets) for the minimum, mean and maximum ADC of the Darkest Part of the Tumor 2D ROI segmentation approach.

**Additional file 3: Figure S3.** ROC curves and AUC (in brackets) for the mean ADC of the Point Tumor ROI segmentation approach.

**Additional file 4: Figure S4.** Scatterplots of concordance correlation coefficients between reader 1 and reader 2 at the Time 1 and at Time 2 regarding whole tumor (WTu) apparent diffusion coefficient (ADC) maximum (A,B), WTu ADC mean (C,D), and WTu ADC minimum (E,F).

**Additional file 5: Figure S5.** Scatterplots of concordance correlation coefficients between reader 1 and reader 2 at the Time 1 and at Time 2 regarding darkest part of the tumor (DpTu) apparent diffusion coefficient (ADC) maximum (A,B), DpTu ADC mean (C,D), and DpTu ADC minimum (E,F).

**Additional file 6: Figure S6.** Scatterplots of concordance correlation coefficients between reader 1 and reader 2 at Time 1 and at Time 2 regarding Point Tumor (Ptu) apparent diffusion coefficient (ADC) mean (A,B).

## Data Availability

The datasets used and/or analyzed during the current study are available from the corresponding author on reasonable request.

## Abbreviations

ADC: Apparent diffusion coefficient
BI-RADS: Breast Imaging Reporting and Data System
DCE: Dynamic contrast-enhanced
DWI: Diffusion-weighted imaging
MRI: Magnetic resonance imaging
NME: Non-mass enhancement
ROI: Region of interest
