# Supplementary material for: Limited role of DWI with apparent diffusion coefficient mapping in breast lesions presenting as non-mass enhancement on dynamic contrast-enhanced MRI
Source: Breast Cancer Res. 2019 Dec 4;21:136. doi: 10.1186/s13058-019-1208-y (PMC6894318; doi:10.1186/s13058-019-1208-y)

Additional File 2

Figure A2: ROC curves and AUC (in brackets) for the minimum, mean and maximum ADC of the Darkest Part of the Tumor 2D ROI segmentation approach.

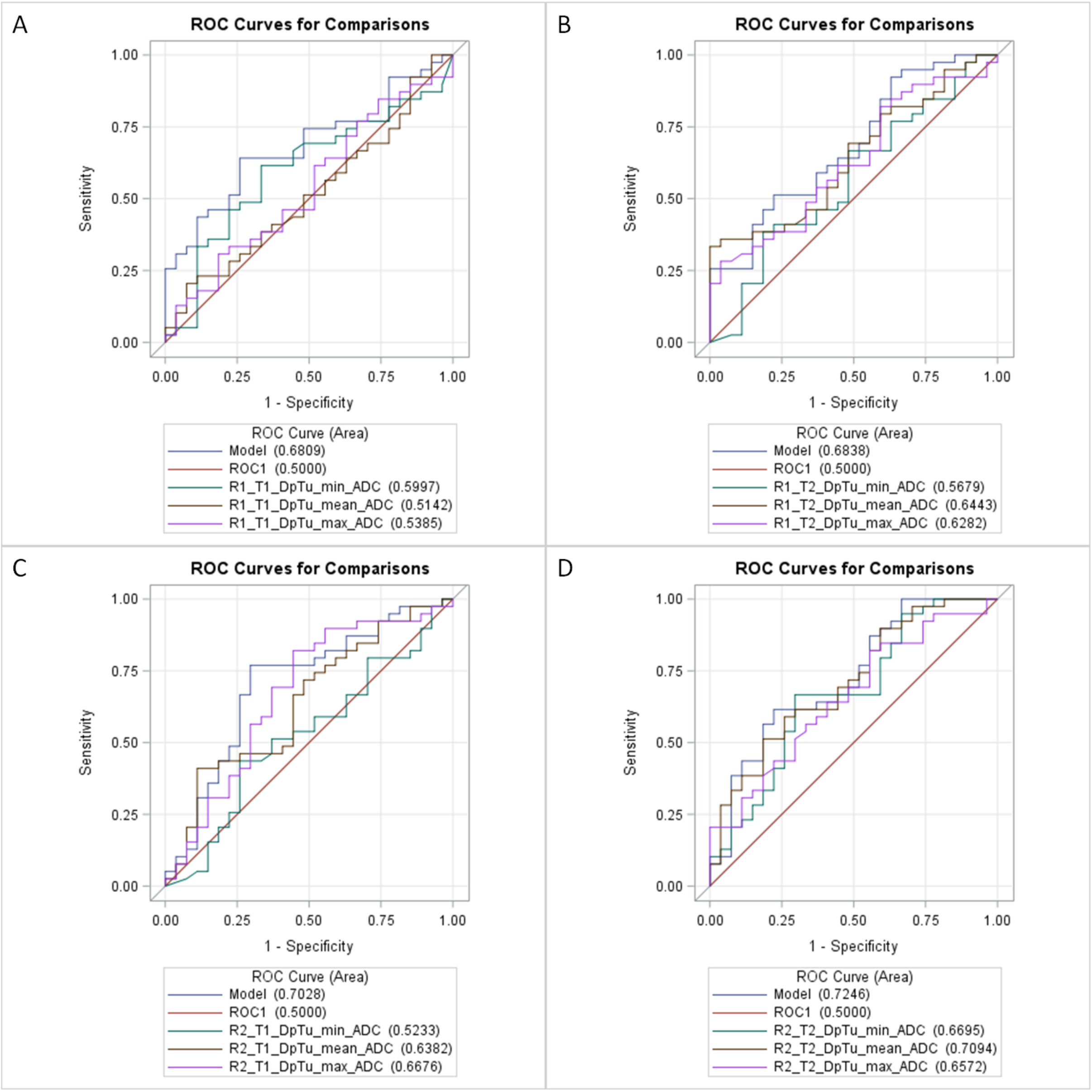

Supplement: Supplementary file 2 — Additional file 2: Figure S2. ROC curves and AUC (in brackets) for the minimum, mean and maximum ADC of the Darkest Part of the Tumor 2D ROI segmentation approach. [file 13058_2019_1208_MOESM2_ESM.pdf]
