# Supplementary material for: Limited role of DWI with apparent diffusion coefficient mapping in breast lesions presenting as non-mass enhancement on dynamic contrast-enhanced MRI
Source: Breast Cancer Res. 2019 Dec 4;21:136. doi: 10.1186/s13058-019-1208-y (PMC6894318; doi:10.1186/s13058-019-1208-y)

Additional File 4

Figure A4: Scatterplots of concordance correlation coefficients between reader 1 and reader 2 at the Time 1 and at Time 2 regarding whole tumor (WTu) apparent diffusion coefficient (ADC) maximum (A,B), WTu ADC mean (C,D), and WTu ADC minimum (E,F).

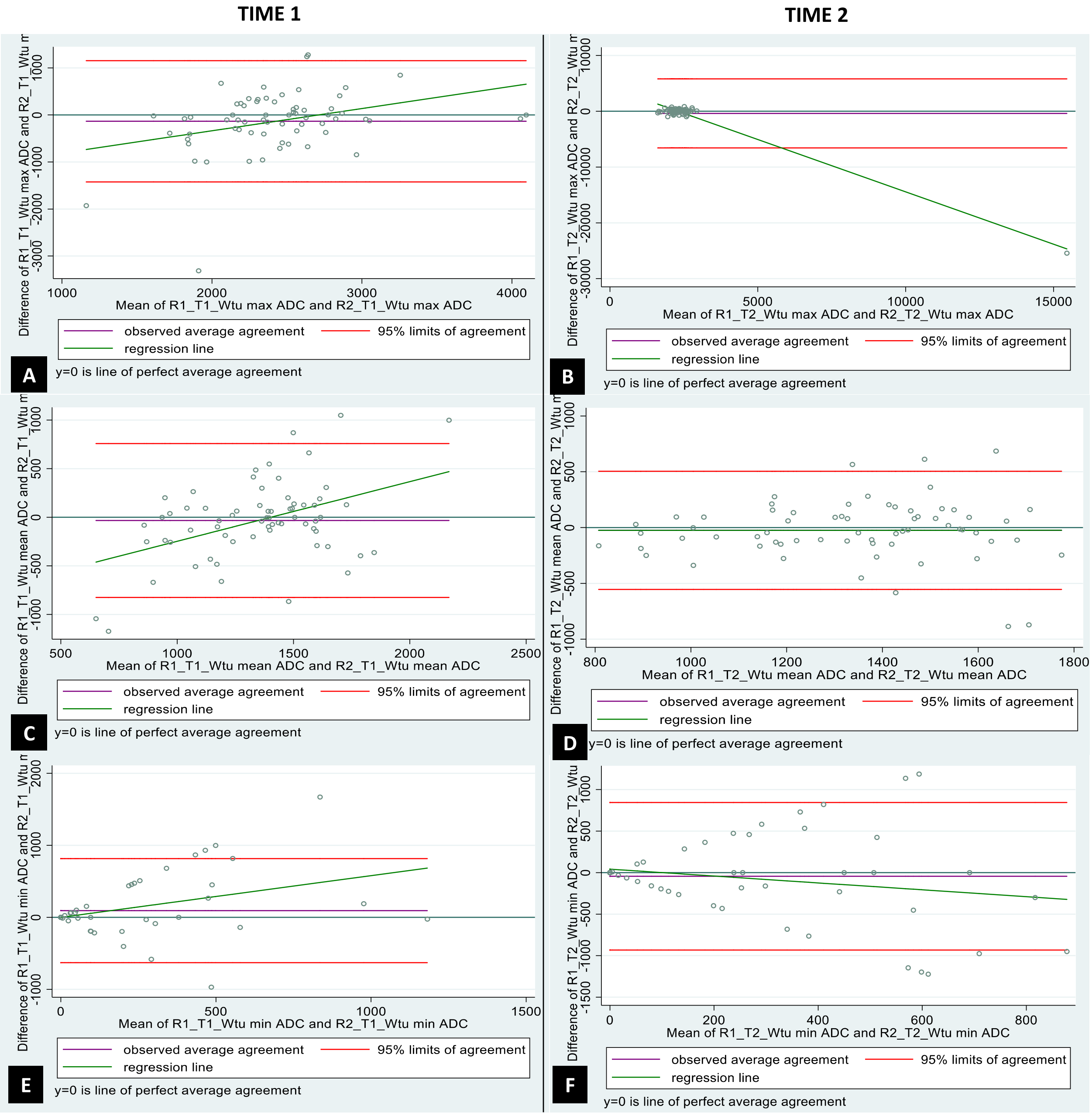

Supplement: Supplementary file 4 — Additional file 4: Figure S4. Scatterplots of concordance correlation coefficients between reader 1 and reader 2 at the Time 1 and at Time 2 regarding whole tumor (WTu) apparent diffusion coefficient (ADC) maximum (A,B), WTu ADC mean (C,D), and WTu ADC minimum (E,F). [file 13058_2019_1208_MOESM4_ESM.pdf]
