# Supplementary material for: Limited role of DWI with apparent diffusion coefficient mapping in breast lesions presenting as non-mass enhancement on dynamic contrast-enhanced MRI
Source: Breast Cancer Res. 2019 Dec 4;21:136. doi: 10.1186/s13058-019-1208-y (PMC6894318; doi:10.1186/s13058-019-1208-y)

**Additional File 5**

**Figure A5:** Scatterplots of concordance correlation coefficients between reader 1 and reader 2 at the Time 1 and at Time 2 regarding darkest part of the tumor (DpTu) apparent diffusion coefficient (ADC) maximum (A,B), DpTu ADC mean (C,D), and DpTu ADC minimum (E,F).

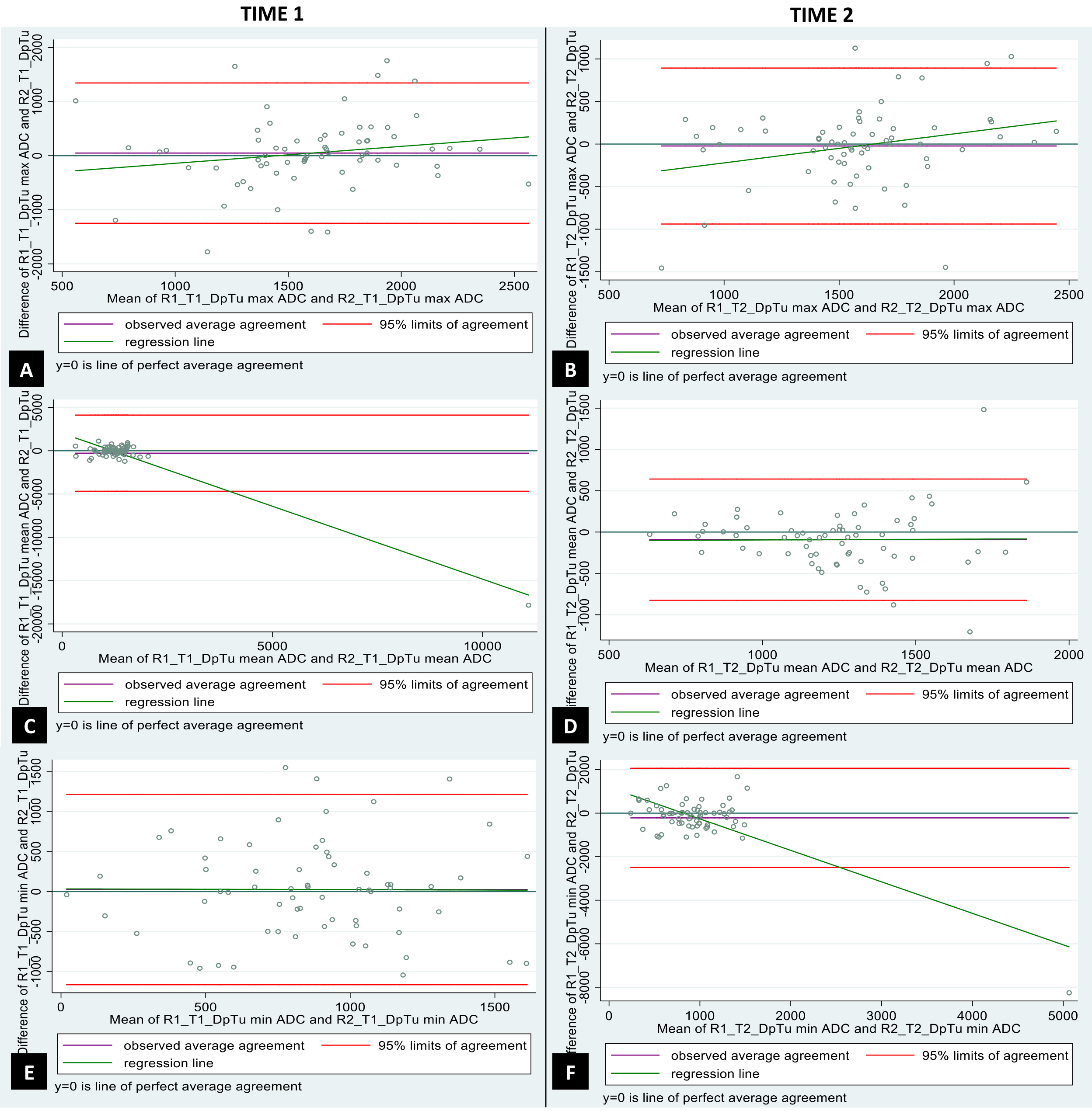

Supplement: Supplementary file 5 — Additional file 5: Figure S5. Scatterplots of concordance correlation coefficients between reader 1 and reader 2 at the Time 1 and at Time 2 regarding darkest part of the tumor (DpTu) apparent diffusion coefficient (ADC) maximum (A,B), DpTu ADC mean (C,D), and DpTu ADC minimum (E,F). [file 13058_2019_1208_MOESM5_ESM.pdf]
