# Supplementary material for: Limited role of DWI with apparent diffusion coefficient mapping in breast lesions presenting as non-mass enhancement on dynamic contrast-enhanced MRI
Source: Breast Cancer Res. 2019 Dec 4;21:136. doi: 10.1186/s13058-019-1208-y (PMC6894318; doi:10.1186/s13058-019-1208-y)

Additional File 6

Figure A6: Scatterplots of concordance correlation coefficients between reader 1 and reader 2 at Time 1 and at Time 2 regarding Point Tumor (Ptu) apparent diffusion coefficient (ADC) mean (A,B).

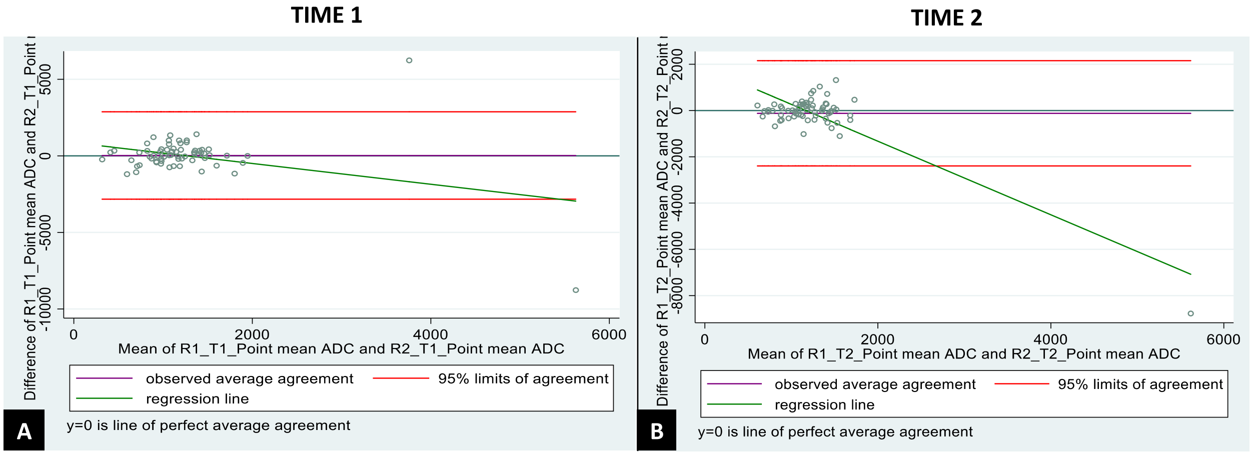

Supplement: Supplementary file 6 — Additional file 6: Figure S6. Scatterplots of concordance correlation coefficients between reader 1 and reader 2 at Time 1 and at Time 2 regarding Point Tumor (Ptu) apparent diffusion coefficient (ADC) mean (A,B). [file 13058_2019_1208_MOESM6_ESM.pdf]
